# Ocular Toxicities of Anticancer Therapies in the Era of Precision Oncology: A Practical, Clinically Oriented Narrative Review

**DOI:** 10.3390/biomedicines14030601

**Published:** 2026-03-08

**Authors:** Fausto Meriggi, Ester Oneda, Sara Cherri, Fausto Petrelli, Alberto Zaniboni

**Affiliations:** 1 Oncology Department, Istituto Ospedaliero Fondazione Poliambulanza, Via Leonida Bissolati, 57, 25124 Brescia, Italy; ester.oneda@poliambulanza.it (E.O.); sara.cherri@poliambulanza.it (S.C.); alberto.zaniboni@poliambulanza.it (A.Z.); 2 Oncology Unit, ASST Bergamo Ovest, Piazzale Ospedale 1, 24047 Treviglio, Italy; fausto_petrelli@asst-bgovest.it

**Keywords:** ocular toxicity, anticancer therapy, targeted therapies, immune-checkpoint inhibitors, antibody–drug conjugates, adverse events

## Abstract

The introduction of modern anticancer therapies, including targeted therapies (TTs), immune checkpoint inhibitors (ICIs), and antibody–drug conjugates (ADCs), has significantly improved survival across a wide range of malignancies. At the same time, these agents have expanded the spectrum of treatment-related adverse events, with ocular toxicities emerging as a clinically relevant and increasingly recognized complication. Ocular adverse events may affect multiple anatomical structures, including the ocular surface, cornea, anterior and posterior segments, and optic nerve, often reflecting drug class-specific biological mechanisms. The pathogenesis of ocular toxicity is multifactorial and includes on-target inhibition of signaling pathways expressed in ocular tissues, off-target effects on rapidly renewing epithelia, non-specific uptake of cytotoxic payloads in ADCs, immune-mediated inflammation associated with ICIs, and microvascular dysregulation observed with selected targeted agents, such as mitogen-activated protein kinase (MEK) inhibitors. Because ocular adverse events are inconsistently reported in clinical trials and frequently described through case reports or pharmacovigilance data, their true incidence is likely underestimated and management strategies remain heterogeneous. This narrative review provides an overview of the epidemiology, biological mechanisms, and clinical manifestations of ocular toxicities associated with contemporary anticancer therapies. In addition, it offers practical, mechanism-based recommendations for prevention, monitoring, and stepwise management, emphasizing the importance of multidisciplinary collaboration to preserve visual function while maintaining effective oncologic treatment.

## Simple Summary

Recent advances in cancer treatment, including targeted therapies, immune checkpoint inhibitors, and antibody–drug conjugates, have substantially improved patient outcomes. However, these therapies are also associated with a growing range of adverse effects, including ocular toxicities that are increasingly encountered in clinical practice. Eye-related side effects are often subtle and nonspecific and may be underestimated or misattributed to aging or systemic illness. Delayed recognition can negatively affect quality of life, lead to treatment modifications, and, in some cases, result in permanent visual impairment. This narrative review summarizes the main ocular toxicities associated with modern anticancer therapies, discusses their underlying biological mechanisms, and provides practical recommendations for prevention, monitoring, and clinical management to support both visual preservation and optimal cancer care.

## 1. Introduction

Over the past two decades, oncology has undergone a profound therapeutic transition. Conventional cytotoxic chemotherapy has been progressively complemented, and in many settings replaced by TTs, immunotherapeutic strategies, and ADCs [1,2,3,4,5,6]. These approaches have transformed outcomes for numerous solid and hematologic malignancies, enabling longer survival and improved disease control [5,6]. At the same time, they have introduced adverse event profiles that differ substantially from those historically associated with cytotoxic agents, including toxicities affecting organs that were previously considered relatively “peripheral” to oncology practice [1,2,3,4,5,6]. The eye is a paradigmatic example [1,2,3,5,6]. Ocular toxicities may be subtle, nonspecific, and easily overlooked, particularly when patients prioritize systemic symptoms or when visual complaints are attributed to aging, fatigue, anemia, or steroid exposure [1,5,6]. Yet, delays in recognition may carry high costs: chronic symptoms that erode quality of life, dose interruptions that compromise oncologic efficacy, and in selected cases, irreversible visual loss [5,6]. These risks are amplified by the fact that ocular events are not uniformly assessed in clinical trials, and available evidence is often derived from retrospective series or pharmacovigilance reports [2,5,6]. Clinically, ocular adverse events span the full anatomic continuum, from ocular surface irritation to posterior segment pathology and neuro-ophthalmic complications [1,2,3,5,6]. Importantly, several drug classes have recognizable patterns: Epidermal Growth Factor Receptor (EGFR) blockade tends to manifest with surface and corneal changes [2,5,6,7,8]; MEK inhibition is linked to serous retinopathy [9,10,11,12]; ICIs can trigger immune-mediated inflammation at multiple ocular levels [13,14,15,16]; and ADCs frequently produce a distinctive corneal phenotype that is often dose-limiting but potentially reversible [17,18,19,20,21,22,23,24,25,26,27,28]. Recognizing these patterns is critical for risk stratification and for tailoring ophthalmic surveillance [5,6,28]. In this narrative review, we provide a clinically oriented overview of ocular toxicities associated with contemporary anticancer therapies. Rather than presenting an exhaustive catalog of rare events, we focus on mechanisms, common phenotypes, and practical approaches to prevention and management aiming to support decision-making at the interface between oncology and ophthalmology [5,6,28]. Estimating the true incidence of ocular toxicities remains challenging. Many events are mild and underreported, while others may be misattributed to unrelated ophthalmic disease [2,5,6]. In pivotal trials, ophthalmologic assessments are often symptom-driven rather than systematic, which biases detection toward more symptomatic presentations [2]. Nevertheless, two consistent messages emerge from the literature and real-world practice [5,6,28]. First, ocular toxicities are not exceptional. They are increasingly observed with novel drug classes, particularly ADCs, where corneal events and visual symptoms commonly lead to dose modification [17,18,19,20,21,22,23,24,25,26,27,28]. Second, severity is heterogeneous. While many patients develop low-grade dry eye, conjunctivitis, or blepharitis [1,2,3,5,6,8], a subset experiences potentially vision-threatening events such as retinal detachment patterns, retinal vascular complications, or optic nerve inflammation, particularly in the context of immune-mediated toxicity [13,14,15,16]. Even “low-grade” ocular surface disease can be clinically meaningful when symptoms are persistent and interfere with reading, driving, or screen exposure, thereby affecting daily functioning and treatment adherence [5,6]. From an oncologic standpoint, ocular toxicity has an additional consequence: it can become a determinant of treatment continuity. In ADC-associated keratopathy, for example, dose delay or reduction is often the most effective intervention to restore tolerability, and may permit ongoing therapy without permanent discontinuation [18,20,21,22,23,24,25,26,27]. This underscores the need for early identification and structured management pathways that preserve both vision and oncologic benefit [5,6,18,21]. The most common intraocular neoplasm is metastatic disease, which most frequently involves the uveal tract. The uvea comprises the iris and ciliary body anteriorly and the choroid posteriorly. Choroidal metastases typically present as elevated, amelanotic chorioretinal lesions with overlying patchy alterations of the retinal pigment epithelium. They are often associated with a serous retinal detachment that is disproportionate to the size of the lesion. Clinical symptoms depend on the location of the metastasis: a peripheral lesion with a limited serous component may be asymptomatic, whereas macular involvement or the presence of subretinal fluid can lead to photopsia (subjective perception of flashes, sparks, or colors), metamorphopsia (distortion of object perception), myodesopsia, blurred vision, difficulty focusing, and dyschromatopsia (altered color perception). Although some of these symptoms may overlap with those of drug-induced ocular toxicity, a dilated fundus examination, often supplemented by ocular ultrasonography and optical coherence tomography (OCT), generally allows choroidal metastases to be distinguished from drug-induced retinopathy. Metastases to the orbit or optic nerve may also occur and can be associated with optic disc edema. Papilledema may likewise be secondary to brain metastases, and cranial imaging is useful for diagnosis in such cases. Retinal metastases can cause inflammation and intraretinal hemorrhages, whereas vitreous metastases lead to an accumulation of leukocytes in the vitreous humor, resulting in a clinical picture consistent with vitritis. A dilated fundus examination and, when indicated, vitreous biopsy can aid in distinguishing true inflammatory vitritis from metastatic infiltration [1].

The eye contains highly specialized tissues with distinct vulnerabilities. The corneal epithelium has rapid cellular turnover and depends on tightly regulated proliferation and migration, making it sensitive to therapies that disrupt epithelial homeostasis or cytoskeletal integrity [2,5,6]. The retina and optic nerve are neurovascular structures vulnerable to inflammatory, ischemic, and toxic–metabolic insults [5,6]. Finally, the eye is characterized by relative immune privilege; therapeutic disruption of immune tolerance, an intended effect of ICIs, can unmask autoimmune inflammation involving ocular tissues [13,14,15,16].

Across anticancer therapies, five broad mechanistic categories can be used to conceptualize ocular toxicities [5,6,29]:

On-target effects in ocular tissues: Targets such as EGFR, Human Epidermal Growth Factor Receptor 2 (HER2), and Mitogen-Activated Protein Kinase (MAPK)-related pathways are expressed in ocular structures. Inhibiting these pathways can disrupt normal ocular physiology [2,5,6].

Off-target exposure and epithelial toxicity: Systemic agents may reach ocular tissues and affect rapidly renewing epithelia, particularly the cornea and conjunctiva [2,5,6].

Payload-driven toxicity (ADCs): Corneal epithelial cells can take up ADC payloads non-specifically, producing characteristic microcystic epithelial changes, often independent of tumor target expression in the eye [17,18,19,20,21].

Immune-mediated inflammation (ICIs): Loss of immune tolerance may lead to uveitis, scleritis, retinitis, vasculitis, or optic neuritis [13,14,15,16].

Microvascular dysregulation: Some targeted agents influence retinal/choroidal fluid dynamics and microcirculation, predisposing to serous retinopathy and, more rarely, vascular occlusive events [9,10,11,12].

This framework is useful because it links the clinical picture to likely pathogenesis and suggests rational management: symptomatic support for surface disease, immunosuppression for immune-mediated events, and exposure reduction (dose modification) when toxicity is driven by cumulative drug or payload exposure [5,6,13,18,21,28].

## 2. Main Pathophysiological Mechanisms

Ocular toxicity associated with ICIs and ADCs arises from the interplay of multiple regulated cell death and cellular stress pathways. In ICI-related ocular inflammation, pyroptosis represents a key mechanism. Activation of the NLRP3 inflammasome triggers caspase-1-mediated cleavage of gasdermin D, leading to membrane pore formation and release of interleukin (IL)-1β and IL-18, thereby amplifying intraocular inflammation and perpetuating immune-mediated tissue injury.

Ferroptosis may play a prominent role in retinal damage. This iron-dependent form of regulated cell death is characterized by uncontrolled lipid peroxidation and impaired antioxidant defenses, particularly reduced glutathione peroxidase 4 (GPX4) activity. Retinal pigment epithelial (RPE) cells and photoreceptors, owing to their high metabolic activity and intrinsic susceptibility to oxidative stress, are especially vulnerable to iron-driven oxidative injury. Experimental evidence supports the central role of oxidative stress and inflammatory signaling in retinal cellular injury, highlighting the contribution of redox imbalance to Müller cell dysfunction and retinal degeneration [29].

Mitochondrial dysfunction further contributes to tissue damage through loss of membrane potential, impaired oxidative phosphorylation, and excessive reactive oxygen species (ROS) generation. ROS accumulation promotes lipid, protein, and DNA damage and may potentiate both inflammasome activation and ferroptotic signaling. Simultaneously, sustained endoplasmic reticulum (ER) stress and prolonged activation of the unfolded protein response (UPR) can shift from adaptive signaling toward pro-apoptotic and pro-inflammatory pathways.

At the ocular surface, corneal epithelial integrity depends on tightly regulated cytoskeletal architecture. Disruption of actin filaments and microtubules compromises epithelial barrier function, adhesion, and regenerative capacity. Corneal epithelial cells internalize circulating therapeutic agents via receptor-independent macropinocytosis and clathrin-mediated endocytosis, mechanisms widely described in nanodrug delivery systems [30]. Following lysosomal trafficking, proteolytic cleavage of ADC linker components results in intracellular release of cytotoxic payloads such as monomethyl auristatin F (MMAF) and DM4, both potent microtubule inhibitors.

Intracellular accumulation of these payloads may induce lysosomal membrane permeabilization, further amplifying cellular stress through cathepsin release and secondary activation of cell death pathways. Concurrent microtubule disruption interferes with mitotic spindle assembly and epithelial turnover, impairing corneal regeneration. Progressive lysosomal overload and dysfunction have therefore been proposed as central mechanisms underlying microcystic epithelial keratopathy, linking impaired intracellular trafficking to epithelial vacuolization, microcyst formation, and loss of corneal transparency, consistent with emerging evidence on lysosome-targeted therapeutic strategies and lysosomal vulnerability in cancer biology [31].

## 3. Clinical Phenotypes Organized by Anatomy

Ocular surface and adnexa are the most common entry points. For many patients, ocular toxicity begins with non-specific irritation: dryness, burning, redness, foreign-body sensation, or tearing [1,2,3,5,6,8]. These symptoms may reflect tear film instability, meibomian gland dysfunction, conjunctival inflammation, or lacrimal drainage impairment. Although often labeled “mild,” chronic ocular surface disease can be disabling, particularly for patients who rely on prolonged screen time or reading [5,6]. EGFR pathway inhibition is a classic driver of ocular surface and eyelid changes, including blepharitis and trichomegaly [2,5,6,7,8]. Cytotoxic agents can also contribute through epithelial injury and, in some cases, nasolacrimal duct obstruction leading to epiphora [1,3,5,6]. Corneal toxicity is clinically important because it directly affects visual acuity and contrast sensitivity. Superficial punctate keratitis may present with photophobia, pain, and fluctuating vision [5,6], while microcystic epithelial keratopathy, particularly associated with several ADCs, often produces blurred vision, halos, and significant functional impairment [17,18,19,20,21,22,23,24,25,26,27]. In ADC-associated corneal toxicity, the phenotype is frequently bilateral and dose-dependent [18,19,20,21,22,23,24,25,26,27]. Importantly, it is often reversible, especially when recognized early and managed with supportive care and treatment modification [18,20,21,22,23,24,25,26,27]. However, repeated exposure and inadequate monitoring may increase the risk of persistent epithelial instability [18,21]. Anterior uveitis and iridocyclitis are hallmark complications of immune modulation. ICIs can induce uveitis at various levels, sometimes bilaterally, and may coexist with other systemic immune-related adverse events. Scleritis and episcleritis, though less common, can be intensely painful and clinically significant. These presentations require timely ophthalmologic evaluation both to preserve vision and to guide systemic treatment decisions [13,14,15,16]. Posterior segment involvement is less frequent than anterior ocular toxicity but is associated with a higher risk of clinically meaningful and potentially irreversible visual impairment, warranting increased clinical awareness in oncology practice [5,6]. Among targeted therapies, MEK inhibitors are classically associated with serous retinal changes, commonly referred to as MEK inhibitor-associated retinopathy (MEKAR). This entity is typically characterized by bilateral subretinal fluid accumulation detectable on optical coherence tomography, often in the absence of significant fundoscopic abnormalities. Onset usually occurs early after treatment initiation and may be transient; however, persistent or recurrent cases have been reported, particularly in patients receiving combination regimens. Patients may present with blurred vision, metamorphopsia, or scotomas, although a substantial proportion remain asymptomatic. This dissociation between symptoms and structural retinal changes supports a low threshold for ophthalmologic referral in patients reporting visual disturbances and suggests a role for structured monitoring in selected high-risk settings, including combined targeted and immunotherapy approaches [9,10,11,12]. In contrast, ICIs are more frequently associated with inflammatory neuro-ophthalmic adverse events, which, although rare, can be severe and vision-threatening. These include optic neuritis, as well as retinal vasculitis and retinal vascular occlusive events, often with acute onset and rapid visual deterioration. Such complications require urgent multidisciplinary evaluation, treatment interruption, and prompt initiation of systemic immunosuppression, most commonly high-dose corticosteroids [13,14,15,16].

For the clinician, it is crucial to identify and to grade the severity of ocular adverse events. The National Cancer Institute’s Common Terminology Criteria for Adverse Events (NCI-CTCAE) system includes severity grading tables for a wide range of ocular conditions, including blurred vision, cataract, conjunctivitis, corneal ulcer, dry eye, extraocular muscle paresis, ocular pain, eyelid motility disorders, photopsias, myodesopsia, glaucoma, keratitis, nyctalopia, optic nerve disorders, papilledema, photophobia, retinal detachment, retinal tear, retinal vascular disorders, retinopathy, scleral abnormalities, uveitis, vitreous hemorrhage, excessive lacrimation, and other ocular toxicities [32] (Table 1).

## 4. Major Ocular Toxicities Across Main Drug Classes (Table 2)

Traditional chemotherapy remains relevant, particularly in combination regimens, including in gynecologic oncology where ocular adverse events are increasingly reported. Its ocular effects largely reflect vulnerability of rapidly dividing cells. Taxanes are associated with tearing and lacrimal drainage problems, whereas antimetabolites can induce keratitis and photophobia. Platinum compounds have rare but serious reports of optic neuropathy. These toxicities are often dose-related and may improve after treatment cessation, but structural complications (e.g., lacrimal obstruction) can persist [1,3,5,6,33].

TTs often produce ocular events that align with pathway biology [2,5,28]. EGFR inhibition can impair epithelial maintenance of the ocular surface, predisposing to dry eye, conjunctivitis, keratitis, and, rarely, corneal ulceration [2,5,6,7,8]. Patients may also develop trichomegaly, a distinctive eyelash change [2,5,6]. B-RAF Proto-Oncogene, Serine/Threonine (BRAF)/MEK inhibition is strongly linked to posterior segment changes, particularly serous retinal detachments and retinopathy patterns. While many cases are manageable and reversible, the potential for significant visual symptoms necessitates awareness and timely ophthalmic evaluation [9,10,11,12]. Fibroblast Growth Factor Receptor (FGFR) inhibitors have been associated with ocular toxicity including retinal and/or corneal findings in trials and safety reports, supporting a role for planned monitoring in susceptible patients and careful counseling regarding early symptom reporting [34,35,36] (Table 3).

ICIs have reshaped cancer outcomes and introduced immune-related adverse events (irAEs) affecting multiple organs, including the eye. Reported ocular irAEs are relatively uncommon but likely underestimated, and may occur early (often within months) or late, even after therapy discontinuation. The clinical spectrum is broad: uveitis is the most commonly described entity, but scleritis, episcleritis, optic neuritis, retinitis, and retinal vasculitis can occur. Autoimmune-like dry eye may mimic Sjögren-like disease and may coexist with systemic sicca symptoms [13,16]. Management is guided by severity and the anatomic site involved, and clinical practice guidelines support a severity-adapted approach and multidisciplinary decision-making [13,14]. Mild surface symptoms may respond to lubrication and topical therapy, whereas uveitis or optic nerve involvement often requires topical and/or systemic corticosteroids and, at times, additional immunosuppression. Decisions regarding ICI interruption or rechallenge should be individualized and made collaboratively, balancing cancer control with the risk of recurrent or progressive ocular inflammation [13,14,15,16].

Among modern therapies, ADCs occupy a central position in ocular toxicity discussions because they frequently produce characteristic corneal findings and are common drivers of dose modification [17,18,19,20,21,22,23,24,25,26,27,28]. The dominant mechanism appears to be non-specific uptake of the cytotoxic payload by corneal epithelial cells, which can occur independently of tumor target expression in ocular tissue [17,18,19,20,21]. Clinically, patients often report blurred vision, photophobia, and foreign-body sensation. Slit-lamp examination may reveal microcystic epithelial keratopathy. These findings are often bilateral and cumulative, and may substantially impair function during therapy. Reassuringly, many cases improve with supportive management and systemic dose modification [18,19,20,21,22,23,24,25,26,27]. This reversibility makes early detection especially valuable [18,21]. Because ADC-related ocular toxicity is relatively predictable, structured protocols have emerged in clinical practice, including baseline evaluation, scheduled follow-up, prophylactic artificial tears, and clear pathways for dose interruption or reduction [18,21,23,25,27]. The preventive value of routine prophylactic topical steroids remains debated and should be individualized under ophthalmology oversight [18,21].

**Table 2:** Major ocular toxicities across main drug classes.

Drug Class	Representative Agents	Ocular Toxicities	Incidence (%)	Ocular Structures Involved	Clinical Notes/Management	Refs
ADCs	Belantamab mafodotin, Mirvetuximab soravtansine, T-DXd	Corneal epitheliopathy, blurred vision, microcysts	25–70%	Corneal epithelium	Dose-limiting; prophylactic eye care recommended	[17,18,19,20,21,22,23,24,25,26,27,28,37]
TTs	Multiple agents	Mixed anterior and posterior segment toxicities	Variable	Cornea, retina, optic nerve	Baseline and follow-up ophthalmic monitoring advised	[5,7,28,38]
ICIs	Nivolumab, Pembrolizumab, Ipilimumab	Uveitis, optic neuritis, VKH-like syndrome	1–4%	Uvea, optic nerve	Immune-mediated; corticosteroids often required	[5,13,14,15,16,39,40]
BRAF/MEK Inhibitors	Trametinib, Cobimetinib	MEK-associated retinopathy, uveitis	20–90%	Retina, macula	Often reversible; ophthalmic surveillance recommended	[9,10,11,12,41]
Chemotherapy Agents	Taxanes, Antimetabolites, Platinum compounds	Epiphora, optic neuropathy, blurred vision	5–25%	Lacrimal system, optic nerve	More frequent with docetaxel; supportive care	[1,3,5,6,33,42]
Breast Cancer Therapies (Overview)	Chemotherapy, targeted, hormonal agents	Dry eye, surface disease, inflammatory events	10–35%	Ocular surface	Chronic but usually mild	[43]
ALK Inhibitors	Crizotinib	Visual disturbances, retinal disorders	10–30%	Retina	Real-world pharmacovigilance data	[44]

Abbreviations. ADCs, Antibody–Drug Conjugates; T-DXd, Trastuzumab Deruxtecan; ICIs, Immune-Checkpoint Inhibitors; TTs, Targeted Therapies; BRAF/MEK, Mitogen-Activated Protein Kinase Kinase/B-RAF Proto-Oncogene, Serine/Threonine; ALK, Anaplastic Lymphoma Kinase; Refs, References.

**Table 3:** Graphical summary of monitoring and management of FGFR inhibitor-related ocular toxicity.

Clinical Domain	Futibatinib [36]	Pemigatinib [35]
Time to onset	Median: ~40 days	Median: ~62 days
Baseline monitoring	Comprehensive ophthalmologic exam + OCT before treatment initiation	Comprehensive ophthalmologic exam + OCT before treatment initiation
Scheduled follow-up	Every 2 months for first 6 months, then every 3 months	Every 2 months for first 6 months, then every 3 months
RPED (asymptomatic/stable)	Continue treatment at current dose with close ophthalmologic surveillance	Continue treatment if asymptomatic and stable on serial examinations
RPED (improving)	If resolution within 14 days, maintain current dose	If asymptomatic and improved, resume at reduced dose
RPED (persistent/worsening)	Withhold treatment until resolution; resume at same or reduced dose	Withhold treatment; consider permanent discontinuation if no improvement

Abbreviations: OCT, optical coherence tomography; RPED, retinal pigment epithelial detachment.

## 5. Practical Approach to Prevention, Monitoring, and Management

A baseline ophthalmologic evaluation is particularly reasonable for patients with pre-existing ocular disease (severe dry eye, corneal disease, glaucoma, uveitis, retinal pathology) [5,6,28] and for patients starting therapies with higher ocular risk profiles (many ADCs, MEK inhibitors, ICIs in contexts where risk is heightened or when combined regimens are planned) [9,10,11,12,13,14,15,16,18,19,20,21,22,23,24,25,26,27]. Baseline assessment should be pragmatic and include: symptom history, visual acuity, slit-lamp examination (including fluorescein staining when indicated), intraocular pressure, and fundus evaluation if posterior involvement is plausible [5,6,28]. Patients should be instructed to report promptly new blurred vision, photophobia, ocular pain, or marked redness [5,6,13,14,15,16], new floaters, scotomas, metamorphopsia, or visual field changes [9,10,11,12], and persistent foreign-body sensation or tearing [5,6]. Early reporting is often the difference between reversible toxicity managed conservatively and advanced disease requiring prolonged interruption or systemic immunosuppression [5,6,13,14,15,16,21,23]. Monitoring should reflect both drug class and risk: ADCs often justify scheduled corneal/surface surveillance because toxicity can be common and dose-dependent [18,19,20,21,22,23,24,25,26,27]. MEK/BRAF inhibitor therapy warrants a low threshold for retinal evaluation with any visual symptoms; high-risk patients may benefit from baseline documentation and follow-up [9,10,11,12]. ICI therapy requires symptom-driven pathways with rapid ophthalmology access; recurrent symptoms or prior ocular irAEs may justify closer follow-up [13,14,15,16].

Management integrates ophthalmic supportive care with systemic therapy decisions [13,16,17,18,19,20]:

- Mild ocular surface disease: preservative-free lubricants, lid hygiene, management of meibomian dysfunction, and topical anti-inflammatory therapies when appropriate [17,18].
- Corneal toxicity (especially ADC-related): lubrication, symptom control, and early consideration of dose delay/reduction when functional impairment emerges; reassessment before rechallenge [13,14,15,16,23,24,25,26,27,28].
- Immune-mediated inflammation (uveitis/scleritis/optic neuritis): ophthalmology-led treatment with topical and/or systemic corticosteroids and consideration of steroid-sparing immunosuppression for refractory cases; oncologic decisions on interruption/rechallenge individualized [13,14,15,16].
- Posterior segment involvement: urgent evaluation; treat the underlying mechanism (immune suppression for inflammatory disease; therapy modification for drug-related retinopathy), and monitor closely for recovery [9,10,11,12,13,14,15,16].

Systematic documentation using standardized toxicity grading (e.g., CTCAE) and reporting to pharmacovigilance systems remain important, particularly given ongoing gaps in guideline standardization [2,5,6,32].

## 6. Conclusions

Ocular toxicities are a growing clinical challenge in modern oncology, driven by the expanding use of TTs, ICIs, and ADCs. Although many events are mild, a meaningful subset can be functionally limiting or vision-threatening, with downstream consequences for quality of life and treatment continuity. A discursive, clinically grounded approach, recognizing drug-class patterns, educating patients, implementing risk-adapted monitoring, and managing toxicity through multidisciplinary collaboration, offers the best pathway to preserve vision without compromising oncologic outcomes. As anticancer therapies continue to evolve, standardized guidance and prospective data will be essential to refine prevention and management strategies. The literature is limited by underreporting, heterogeneity in definitions and grading, and the scarcity of prospective ophthalmic surveillance in oncology trials. Mechanistic understanding remains incomplete for several agents, and long-term outcomes after prolonged therapy are not well characterized. Future work should prioritize prospective cohorts with standardized ophthalmic endpoints, development of shared clinical algorithms, and identification of predictive biomarkers that enable risk-adapted monitoring and personalized preventive strategies.

## Figures and Tables

**Table 1:** Ocular adverse events graded according to NCI CTCAE v6.0 [32].

CTCAE Term (Eye Disorders)	Grade 1	Grade 2	Grade 3	Grade 4
Blurred vision	Intervention not indicated	Symptomatic; moderate ↓VA (BCVA ≥ 20/40 or ≤3 lines ↓); limiting instrumental ADL	Marked ↓VA (BCVA < 20/40 or >3 lines ↓ up to 20/200); limiting self-care ADL	BCVA ≤ 20/200
Cataract	Asymptomatic; observation only	Symptomatic; moderate ↓VA and/or glare; limiting instrumental ADL	Marked ↓VA; limiting self-care ADL	BCVA ≤ 20/200
Corneal ulcer	—	—	Corneal ulcer without perforation	Perforation
Decreased night vision	Symptomatic, not limiting ADL	Moderate ↓VA; limiting instrumental ADL	Marked ↓VA; limiting self-care ADL	BCVA ≤ 20/200
Dry eye	Findings only; relieved by lubricants	Symptomatic; moderate ↓VA	Marked ↓VA; limiting self-care ADL	—
Extraocular muscle paresis	Asymptomatic; observation only	Unilateral paresis without diplopia	Bilateral paresis or unilateral with diplopia in peripheral gaze	Diplopia in central gaze or head turning required
Eye pain	Mild pain	Moderate pain; limiting instrumental ADL	Severe pain; limiting self-care ADL	—
Eyelid function disorder	Observation only	Symptomatic; non-operative intervention	Operative intervention indicated	—
Flashing lights	Symptomatic	Limiting instrumental ADL	Limiting self-care ADL	—
Floaters	Symptomatic	Limiting instrumental ADL	Limiting self-care ADL	—
Glaucoma	<8 mmHg elevated IOP	Topical therapy indicated	Visual field defects	Central visual field loss
Keratitis	Asymptomatic; observation only	Symptomatic; moderate ↓VA	Marked ↓VA and/or corneal ulcer	Perforation and/or BCVA ≤ 20/200
Optic nerve disorder	Asymptomatic; observation only	Moderate ↓VA	Marked ↓VA (up to 20/200)	BCVA ≤ 20/200
Papilledema	Asymptomatic	Symptomatic; moderate ↓VA	Marked ↓VA	BCVA ≤ 20/200
Periorbital edema	Soft, non-pitting	Indurated or pitting; topical therapy	With visual disturbance or ↑IOP	—
Photophobia	Symptomatic	Limiting instrumental ADL	Limiting self-care ADL	—
Retinal detachment	—	—	Macula-on detachment	Macula-off detachment
Retinal tear	No detachment; observation	Treatment indicated	—	—
Retinal vascular disorder	—	Without neovascularization	With neovascularization	—
Retinopathy	Asymptomatic; observation only	Symptomatic; moderate ↓VA	Marked ↓VA; limiting self-care ADL	BCVA ≤ 20/200
Scleral disorder	No change in vision	Symptomatic; moderate ↓VA	Marked ↓VA; limiting self-care ADL	BCVA ≤ 20/200
Uveitis	Anterior uveitis with trace cells	Anterior uveitis with 1 + −2+ cells	≥ 3+ cells or intermediate/posterior/panuveitis	BCVA ≤ 20/200
Vision decreased	—	Moderate ↓VA (BCVA ≥ 20/40 or ≤ 3 lines ↓)	Marked ↓VA (up to 20/200)	BCVA ≤ 20/200
Vitreous hemorrhage	Intervention not indicated	Symptomatic; moderate ↓VA	Marked ↓VA and/or vitrectomy indicated	BCVA ≤ 20/200
Watering eyes	Intervention not indicated	Symptomatic; moderate ↓VA	Marked ↓VA	BCVA ≤ 20/200
Eye disorders—Other, specify	Asymptomatic; observation only	Intervention indicated; limiting instrumental ADL	Not immediately sight-threatening; limiting self-care ADL	Sight-threatening; urgent intervention

Abbreviations: CTCAE, Common Terminology Criteria for Adverse Events; BCVA, Best Corrected Visual Acuity; VA, Visual Acuity; ADL, Activities of Daily Living; IOP, Intraocular Pressure; ↓, decrease. Grading according to NCI CTCAE version 6.0 [32].

## Data Availability

No new data were created or analyzed in this study.
